# Protective Effect of Danhong Injection on Acute Hepatic Failure Induced by Lipopolysaccharide and D-Galactosamine in Mice

**DOI:** 10.1155/2014/153902

**Published:** 2014-03-17

**Authors:** Ying Wang, Li-Na Gao, Yuan-Lu Cui, Heng-Li Jiang

**Affiliations:** Tianjin State Key Laboratory of Modern Chinese Medicine, Research Center of Traditional Chinese Medicine, Tianjin University of Traditional Chinese Medicine, No. 88 Yuquan Road, Nankai District, Tianjin 300193, China

## Abstract

Acute hepatic failure (AHF), which leads to an extremely high mortality rate, has become the focus of attention in clinic. In this study, Danhong injection (DHI) was investigated to evaluate the preventive and protective effect on AHF induced by lipopolysaccharide (LPS) and D-galactosamine (GalN) in mice. For AHF induction, ICR mice were intraperitoneally injected with D-GalN (700 mg/kg) and LPS (20 **μ**g/kg). DHI was administrated twice, at 12 and 1 h, respectively, before D-GalN/LPS injection. After stimulation with D-GalN/LPS for 1 and 6 h, serum and livers were collected for analysis. We found that mice administrated with DHI displayed a higher survival rate, lower serum levels of alanine aminotransferase (ALT), aspartate aminotransferase (AST), total bilirubin (TBil), glutathione S-transferase (GST), and tumor necrosis factor (TNF)-**α**. DHI inhibited the elevations of hepatic lipid peroxidation (malondialdehyde), caspase-8 activity, and mRNA expression levels of inflammatory cytokines (interleukin-1**β** and interleukin-6) increased by D-GalN/LPS in the liver. Furthermore, liver histopathological analysis indicated that the DHI group showed markedly fewer apoptotic (TUNEL positive) cells and less pathological changes than those in the AHF model group. These results provide a novel insight into the pharmacological actions of DHI as a potential candidate for treating AHF.

## 1. Introduction

Acute hepatic failure (AHF) has become the focus of attention for researchers because of its extremely high mortality rate and the lack of ideal treatment in clinical medicine. AHF is a serious type of hepatitis accompanied by hepatic encephalopathy, which causes multiorgan failure resulting in extremely high mortality [1]. The deterioration of AHF urgently needs to be effectively alleviated. Though many AHF therapies have been proposed and applied in recent years, only hepatic transplantation is widely accepted among clinical specialists. Unfortunately, the application of orthotopic liver transplantation has remained low because of its high expenses and insufficient organ donation. For these reasons, other therapeutic interventions, especially drug therapy, have to be considered [2].

Multiple pathogenic factors are implicated in the pathogenesis of AHF, and some studies have focused on inflammation-associated bacterial toxins, cytokines, oxygen free radicals, and hepatocyte regeneration [3, 4]. Nevertheless, other researchers have shown that hepatocellular apoptosis, which functions as a signal for leukocyte migration and attacking of parenchymal cells, represents a crucial step in AHF. This leads to a vicious circle with aggravation of leukocyte inflammatory reactions and even necrosis. Therefore, pleiotropic agents, exerting multiple functions, may be of particular interest because of their broad potential to interfere with relevant pathways of disease.

For the study of AHF, a classical animal model achieved by intraperitoneal injection of D-galactosamine (GalN) with lipopolysaccharide (LPS) has often been used. D-galactosamine acts as a sensitizing agent because it specifically and reversibly depletes hepatic stores of uridine triphosphate via the galactose pathway. Consequently, serious metabolic alterations and hepatic necrosis immediately occur [2]. Bacterial LPS (endotoxin), a vital trigger of sepsis, is the major component of the outer membrane of Gram-negative bacteria. High doses of LPS induce fever, hypotension, intravascular coagulation, and even multiorgan failure [5]. Low doses of LPS cause modest inflammatory responses resulting in increased susceptibility to numerous hepatotoxic chemicals, such as GalN [6]. As a response, hepatocytes strive to clear LPS, which is followed by inflammation, hepatocellular apoptosis, and even hepatic necrosis.

In the model of AHF induced by D-GlaN/LPS, mortality is widely accepted as a severe result of hepatic damage [7–12]. Furthermore, symptoms, including an increase in liver enzymes, bilirubin, ammonium, or lactate in blood, are similar to the characteristics of AHF patients in the clinic. Therefore, this model accurately reproduces the pattern of human AHF.

Danhong injection (DHI), a Chinese materia medica standardized product, is manufactured from the root and rhizome of* Salvia miltiorrhiza* Bge. (Labiatae, officially recognized in the Chinese Pharmacopoeia as* Salviae miltiorrhizae* Radix et Rhizoma) and the flower of* Carthamus tinctorius* L. (Compositae, officially recognized in the Chinese Pharmacopoeia as Carthami Flos). DHI is applied for activating circulation of the blood and resolving stasis to promote regeneration [13] and is mainly used to treat ischemic encephalopathy and coronary heart disease in clinic [14–16]. Recently, we have reported that DHI could be used for systemic acute inflammatory reaction [17], and we replicated the classical model of AHF in this study to further observe the preventive and protective effect of DHI on AHF. Moreover, we attempted to explore its possible mechanisms in the prophase of liver failure.

## 2. Materials and Methods

### 2.1. Chemicals and Reagents

DHI was purchased from Shandong Buchang Pharmaceutical Co., Ltd (Jinan, China) (drug approval number: Z20026866). LPS from* Escherichia coli *0111:B4 was purchased from Sigma-Aldrich Co. (St. Louis, MO, USA). D-galactosamine hydrochloride (GalN, 98%) and N-acetyl-L-cysteine (NAC, 98%) were purchased from Alfa AesarCo. (Tianjin, China). Detection kits for alanine aminotransferase (ALT), aspartate aminotransferase (AST), glutathione S-transferase (GST), and total bilirubin (TBil) were purchased from Nanjing Jiancheng Bioengineering Institute (Nanjing, China). The lipid peroxidation malondialdehyde (MDA) assay kit was purchased from Beyotime Institute of Biotechnology (China). Tumor necrosis factor (TNF)-*α* Mouse ELISA Kit was obtained from e-Bioscience Co. (USA). The DeadEnd Colorimetric TUNEL System and ImProm-II Reverse Transcription System were purchased from Promega Co. (USA). The Mammalian Cell Lysis Kit and UNIQ-10 column Trizol total RNA extraction kit were bought from Sangon Biological Engineering Technology & Services Co., Ltd. (Shanghai, China). The FastStart Universal SYBR Green Master (ROX) kit was purchased from Roche (Mannheim, Germany).

### 2.2. Preparation and Quality Control Standard of DHI

The quality control standard of DHI conforms to the National Drugs surveillance administrative bureau standard, which regulates that the total amount of danshensu (molecular formula: C_9_H_10_O_5_) and protocatechuic aldehyde (molecular formula: C_7_H_6_O_3_) should not be lower than 0.5 mg in a 1 mL injection, as analyzed by high performance liquid chromatography. Simultaneously, the total flavonoids determined by visible spectrophotometry should not be lower than 5.0 mg/mL against rutin (molecular formula: C_27_H_30_O_16_) [14].

To guide the rational use of DHI in clinic, more and more researchers focus on the fingerprint analysis of DHI and show that more than ten components have been determined such as 5-hydroxymethyl-2-fulfural, danshensu, ferulic acid, rosmarinic acid, lithospermic acid, protocatechuic aldehyde, caffeic acid, salvianolic acid A, salvianolic acid B, and salvianolic acid D [18, 19].

### 2.3. Animals

Male ICR mice (20–22 g) were obtained from Tianjin Experimental Animal Center. To adapt to the experimental environment, mice were maintained in our animal facility with free access to standard chow and water for 7 days before experimentation. Mice were housed collectively in cages measuring 45 × 24 × 20 cm (5 mice per cage) under a 12 : 12 h light/dark cycle (lights off at 7:00 p.m.) in a temperature-controlled room (22 ± 2°C). All of the animals used in this study were in accordance with the NIH Guide for the Care and Use of Laboratory Animals and the protocol was approved by the Ethics Committee of Tianjin University of Traditional Chinese Medicine (TCM-2009-037-E09).

### 2.4. Experimental Design

Before the administration of D-GalN/LPS, animals were fasted overnight (12–16 h) but given access to water ad libitum. In this study, we performed three parallel experiments on animals, to analyze survival rate within 24 h, and other indexes at 1 and 6 h, respectively. According to the clinical dosage (0.3–0.6 mL/kg, for people), we calculated that the experimental dosage for mice was about 3–6 mL/kg which was below the LD_50_ (39.5 mL/kg) [20]. Because we aimed to investigate the preventive and protective effect of DHI on AHF, we chose concentrations including 6, 3, and 1.5 mL/kg for study. Mice were divided into 6 groups (10 mice per group for survival study and 8 mice per group for other experiments) including blank control group, D-GalN/LPS-treated group, D-GalN/LPS + DHI (1.5, 3 and 6 mL/kg), and D-GalN/LPS +* N*-acetyl-L-cysteine (NAC) group (positive control group).* N*-acetyl-L-cysteine (NAC), an antioxidant, has been proved to reduce generation of ROS, activity of caspase-3 and caspase-9, and prevent the liver injury from injury induced by Fas agonist [21]. DHI (1.5, 3 and 6 mL/kg) or NAC (300 mg/kg) was injected i.p. twice, at 12 and 1 h, respectively, before D-GalN/LPS treatment [22, 23]. Mice in the blank control group and D-GalN/LPS-treated group were also given the same volume of phosphate-buffered saline (PBS) for two times. With that, in addition to those in the blank control group which were given the same volume of PBS, other mice were injected intraperitoneally (i.p.) with D-GalN (700 mg/kg) and LPS (20 *μ*g/kg) dissolved in PBS. Mice were killed by rapid decapitation at different time points (1 and 6 h after D-GalN/LPS treated), and then serum and liver were collected. For those sacrificed at 1 h after D-GalN/LPS treated, serum and liver collected were used to investigate the anti-inflammatory effect of DHI on AHF and mice sacrificed at 6 h were prepared to detect antioxidant and antiapoptotic activities.

### 2.5. Measurement of Survival Rate

The survival rate of mice was monitored for 24 h after D-GalN/LPS injection. Mice were divided into groups including blank control group, D-GalN/LPS-treated group, D-GalN/LPS + DHI (1.5, 3 and 6 mL/kg), and D-GalN/LPS + NAC group and administrated with DHI or NAC twice, at 12 and 1 h, respectively, before GalN/LPS injection. The survival rate was calculated from the number of animals that survived by the total number of tested animals at the given time.

### 2.6. Hepatic Lipid Peroxidation, Serum Biochemical, and TNF-*α* Assays

MDA in liver homogenates and ALT, AST, GST, TBil (collected at 6 h after injection with D-GalN/LPS), and TNF-*α* (collected at 1 h after treatment with D-GalN/LPS) in serum were determined using commercial kits.

### 2.7. Real-Time RT-PCR Analysis for mRNA Expression of Interleukin (IL)-1*β* and IL-6 in Mouse Liver

To analyze IL-1*β* and IL-6 mRNA transcription levels in the liver, mice were killed 1 h after D-GalN/LPS injection. Total RNA was extracted using the Sangon UNIQ-10 column Trizol total RNA extraction kit (Shanghai, China) according to the manufacturer's instructions. Reverse transcription was performed with ImProm-II Reverse Transcription System cDNA synthesis kit. The real-time RT-PCR oligonucleotide primers used were as follows: 5′-TGT TAC CAA CTG GGA CGA CA-3′ and 5′-GGG GTG TTG AAG GTC TCA AA-3′ forward and reverse primers for mouse *β*-actin (NM_007393.3); 5′-GAC CTT CCA GGA TGA GGA CA-3 and 5′-AGC TCA TAT GGG TCC GAC AG-3′ forward and reverse primers for mouse IL-1*β* (NM_008361.3); 5′-TCC AGT TGC CTT CTT GGG AC-3′ and 5′-GTG TAA TTA AGC CTC CGA CTT G-3′ forward and reverse primers for mouse IL-6 (NM_031168.1). The reactions were set up in duplicate in 25 *μ*L total volumes with 0.5 *μ*L of each primer (0.3 *μ*M final concentrations), 12.5 *μ*L of FastStart Universal SYBR Green Master (ROX, Roche), and 1 *μ*L of template cDNA. The PCR cycle was as follows: 95°C for 10 min, 40 cycles of 95°C for 15 s, and 60°C for 1 min. Melt curve analysis was performed at the end of each experiment to verify that a single product per primer pair was amplified. The amplification and analysis were performed using an ABI Prism 7500 Real-time PCR System. Samples were compared using the relative threshold cycle (C_*T*_) method. The fold increase or decrease was determined relative to a blank control after normalizing to a housekeeping gene using the 2^−ΔΔC_*T*_^ method [24, 25].

### 2.8. Determination of Caspase-8 Activity

Caspase-8 activity was determined in liver homogenates at 6 h after D-GalN/LPS injection using the commercial caspase-8 colorimetric assay kit (Applygen Technologies Co. Ltd., Beijing, China). After incubation for 0 and 2 h, absorbance was measured at 405 nm. However, the value of OD, calculated as caspase protease activity, represented deviation between OD_2 h_ and OD_0 h_, rather than a direct absorbance.

### 2.9. Hematoxylin and Eosin (H&E) Staining and Terminal Deoxyribonucleotidyl Transferase-Mediated dUTP-Digoxigenin Nick End Labeling (TUNEL) Staining

For histological analysis, liver tissues (6 h after D-GalN/LPS stimulation) were fixed in 10% neutral-buffered formalin and then were embedded in paraffin. Sections 5 *μ*m in thickness were fixed to slides, deparaffinized, and stained with hematoxylin and eosin to determine morphological changes. Slides were examined and photographed under a light microscope (Olympus, Tokyo, Japan) at 400x magnification. Apoptotic cells were detected by TUNEL staining with a commercially available kit (DeadEnd Colorimetric TUNEL System; Promega Corporation, USA).

### 2.10. Statistical Analysis

All results are reported as the mean ± SEM. The overall significance of the data was examined by one-way analysis of variance. Differences between groups were considered significant at *P* < 0.05 and *P* < 0.01 with the appropriate Bonferroni correction made for multiple comparisons.

## 3. Results

### 3.1. Effects of DHI on Survival Rate of Mice

**Figure 1:**
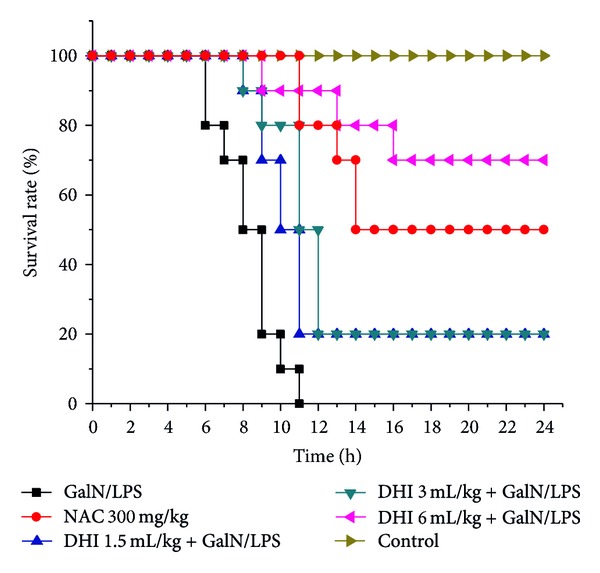
Effects of DHI on the survival rate of mice. Mice were subjected to sterile PBS or DHI (1.5, 3, and 6 mL/kg) at 12 and 1 h before D-GalN/LPS injection. Then, D-GalN (700 mg/kg)/LPS (20 *μ*g/kg) was intraperitoneally injected to mice. The survival rate was assessed for 24 h.

The survival rate of mice was shown in Figure 1 which showed that mice began to die 6 h after D-GalN/LPS administration. Finally, all mice not given DHI died within 11 h. However, in those treated with DHI (1.5, 3 and 6 mL/kg body weight), the survival rate increased by 20%, 20%, and 70%, respectively, 24 h after D-GalN/LPS administration. A high concentration (6 mL/kg) of DHI showed the best protective effect compared with NAC (50% at 24 h). These results showed that DHI inhibited D-GalN/LPS-induced mortality and this inhibition was dose dependent.

### 3.2. Liver Histopathology

The mice in the control group (Figure 3(a)) showed intact lobular architecture and normal structure of hepatocytes. Histological examination of livers exposed to D-GalN/LPS (Figure 3(b)) showed severe liver damage, including massive necrosis with broad hemorrhagic necrosis, intralobular hemorrhage, congestion, and an increase in inflammatory cell infiltration. However, these pathological changes were ameliorated in animals receiving DHI pretreatment, with only slight degeneration and spotty necrosis found in the livers of DHI-treated mice (Figures 3(d)–3(f)). TUNEL staining showed that administration with DHI could significantly ameliorate hepatic cell apoptosis (Figure 4).

### 3.3. Serum Levels of ALT, AST, and GST Activity, TBil Concentrations, and Hepatic Lipid Peroxidation

Serum levels of ALT, AST, and GST activity and TBil concentrations were analyzed to evaluate liver injury. The serum level of ALT (40.9-fold, *P* < 0.01), AST (2.6-fold, *P* < 0.05), TBil (2.5-fold, *P* < 0.05), and GST (1.7-fold, *P* < 0.05) activity were significantly higher in D-GalN/LPS treated rats compared to those in control groups and this increase was suppressed by injection of DHI. The administration of D-GalN/LPS increased hepatic MDA (5-fold, *P* < 0.05) levels compared with those in the control group, but this elevation was attenuated by DHI treatment (Table 1).

### 3.4. Effects of DHI on Serum TNF-*α* Levels and Hepatic Caspase-8 Activity

**Figure 2:**
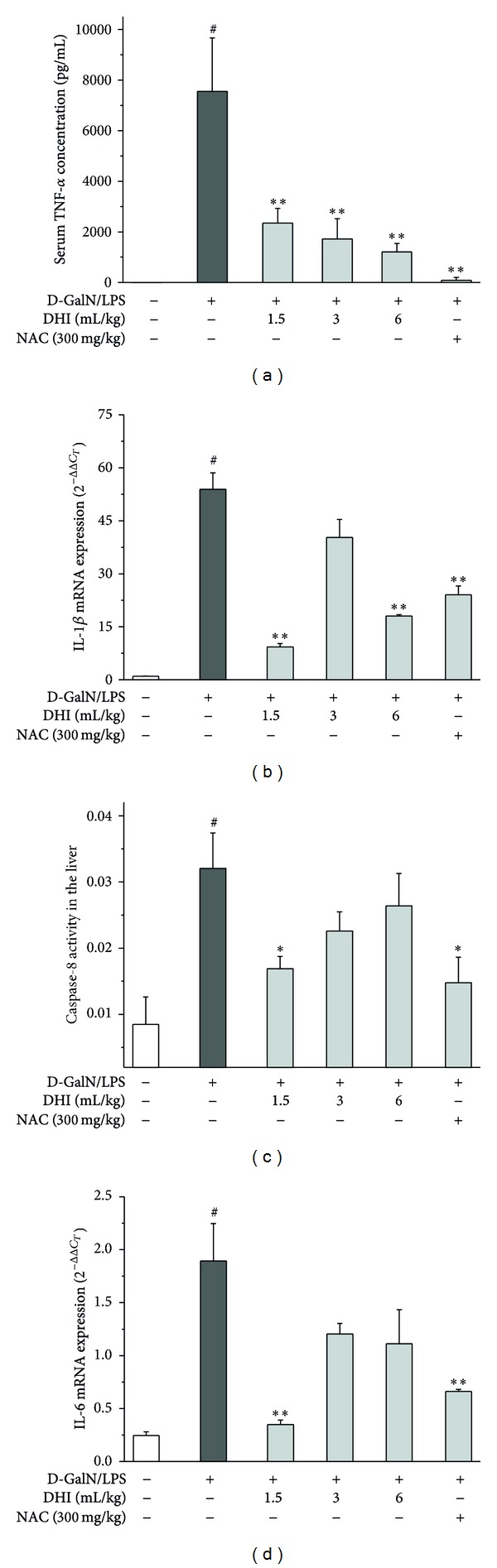
Effects of DHI on serum TNF-*α* levels, caspase-8 activity, and inflammatory mediators expressed in liver. Serum for measuring TNF-*α* level (a) was collected at 1 h after D-GalN/LPS injection. The mRNA expressions of IL-1*β* (b) and IL-6 (d) were analyzed by real-time RT-PCR. Caspase-8 (c) activity was determined in liver homogenates at 6 h after D-GalN/LPS injection using the commercial caspase-8 colorimetric assay kit. Data show mean ± SEM (*n* = 8). ^†^ *P* < 0.05 versus the control group; *P < 0.05 and **P < 0.01 versus the GalN/LPS group.

**Figure 3:**
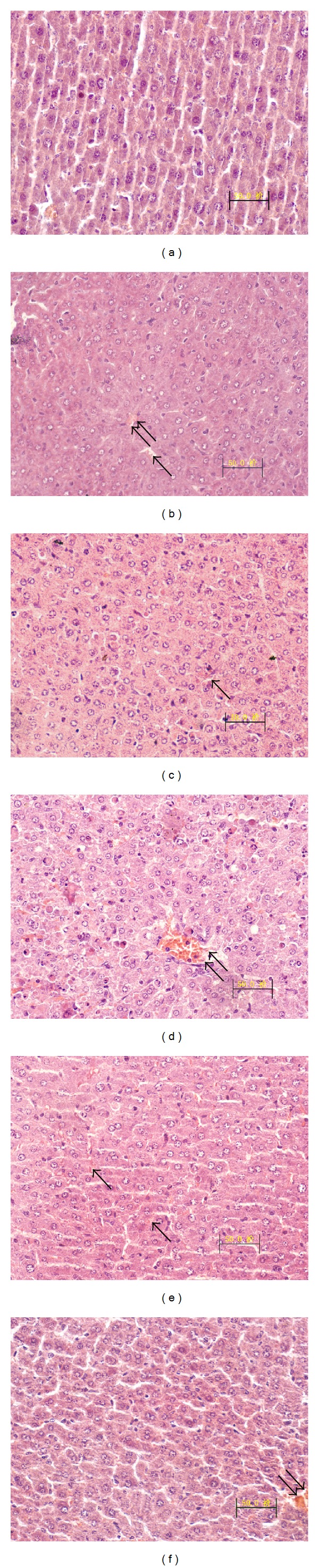
Effects of DHI on the liver. Mice were treated with D-GalN/LPS with or without DHI. Representative images are shown for the different experimental groups (original magnification, 400x). (a) Control group, (b) D-GalN/LPS-stimulated group, (c) NAC-treated group, (d) DHI (6 mL/kg) + D-GalN/LPS-stimulated group, (e) DHI (3 mL/kg) + D-GalN/LPS-stimulated group, and (f) DHI + D-GalN/LPS-stimulated (1.5 mL/kg) group.

**Figure 4:**
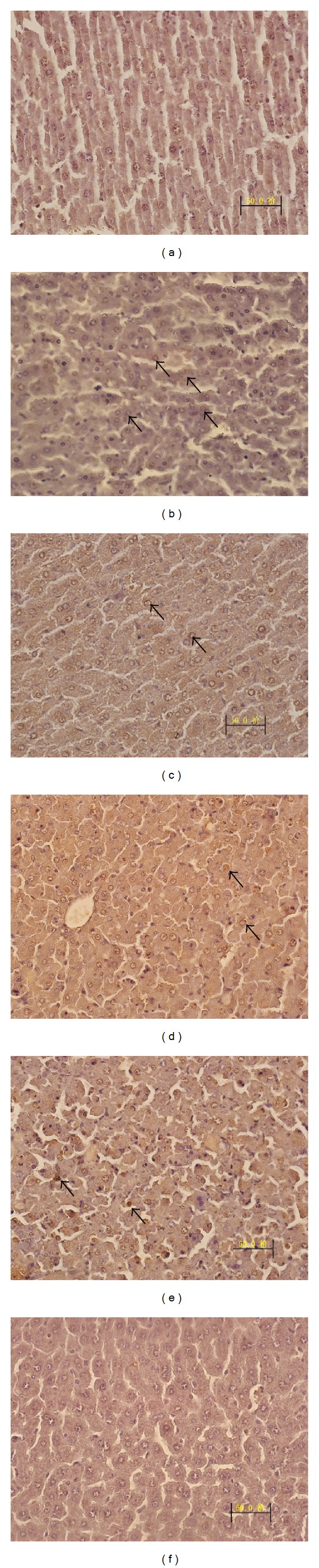
Effects of DHI on apoptosis of hepatocytes. TUNEL assay of apoptotic hepatocytes in the liver was performed 6 h after D-GalN/LPS injection with or without DHI. DHI (1.5, 3, and 6 mL/kg body weight) or sterile PBS was intraperitoneally administrated at 12 and 1 h before D-GalN/LPS injection (original magnification, 400x). (a) Control group, (b) D-GalN/LPS-stimulated group, (c) NAC-treated group, (d) DHI (6 mL/kg) + D-GalN/LPS-stimulated group, (e) DHI (3 mL/kg) + D-GalN/LPS-stimulated group, and (f) DHI + D-GalN/LPS-stimulated (1.5 mL/kg) group.

To further investigate hepatoprotective effects of DHI, serum inflammatory cytokine levels and hepatic caspase-8 activity were measured. As shown in Figure 2(a), DHI (1.5, 3, and 6 mL/kg) significantly reduced the increase of TNF-*α* level in serum induced by D-GalN/LPS injection (*P* < 0.01). However, only DHI (1.5 mL/kg) exerted significant decrease on caspase-8 activity after D-GalN/LPS injection (*P* < 0.05) (Figure 2(c)).

### 3.5. Effect of DHI on D-GalN/LPS-Induced IL-1*β* and IL-6 mRNA Expression

As shown in Figures 2(b) and 2(d), D-GalN/LPS markedly increased the mRNA expression of IL-1*β* and IL-6 (*P* < 0.05), comparing with blank control group. However, DHI inhibited the mRNA expressions of IL-1*β* and IL-6 with different degrees.

## 4. Discussion

DHI has been frequently used in the treatment of cardiovascular and cerebrovascular disease, especially stroke induced by high blood viscosity since 2002 [14, 26]. However, there are no previous reports on the effect of DHI on hepatic protection. In the current study, we found that pretreatment with DHI prevented D-GalN/LPS-induced AHF in mice. In detail, death occurred 6 h after D-GalN/LPS treatment, and the rate of death in the AHF model group rapidly increased to 100% at 11 h. However, administration with DHI reduced lethality by 30%, which indicated that DHI was protective against D-GalN/LPS intoxication.

Transaminase (ALT and AST), GST, MDA, and TBil are important indexes to diagnose and measure the level of hepatic injury in clinic [27]. In the current study, we observed that D-GalN/LPS injection in the AHF model group significantly increased the levels of ALT, AST, GST, TBil, and MDA [28], but attenuated by DHI with different degrees which suggest that DHI may be a potential hepatic protection and antioxidative drug.

In the process of AHF, immune reactions are the first to respond to protect organisms by releasing proinflammatory cytokines [29]. Once excessive inflammatory mediators are released, they in turn attack hepatocytes [30, 31]. Controlling the activation of TNF-*α* is important for protecting against hepatic damage [32]. Interestingly, DHI could significantly decrease the increased TNF-*α* level induced by D-GalN/LPS which suggests that DHI possesses an anti-inflammatory property in protecting liver injury. Activation of TNF-*α* is critically governed by the transcription factor nuclear factor kappa B (NF-*κ*B) [32–34].* Salviae miltiorrhizae* has been proved to possess significant protective effects on apoptosis and NF-*κ*B protein expression of the intestinal mucosa in rats with severe acute pancreatitis or obstructive jaundice [35, 36]. This suggests that DHI protected mice from a serious inflammatory response and may be involved in the signaling pathway of NF-*κ*B. Further study showed that DHI could ameliorate the increased mRNA expressions of inflammatory cytokines IL-1*β* and IL-6 induced by D-GlaN/LPS. Liver staining simultaneously showed numerous inflammatory cell infiltrations, such as mononuclear macrophages in the AHF model group. Therefore, we propose that inflammatory stress might be involved in the development of AHF.

Caspase-8 belongs to the most upstream caspase in the death receptor apoptosis pathway and is directly activated by TNF-*α*. In this study, a marked increase in caspase-8 activity was observed after D-GalN/LPS administration, which indicated that apoptosis was involved in D-GalN/LPS-induced hepatocyte death. However, administration with DHI could block apoptosis of hepatocytes by decreasing the activation of caspase-8 in the liver. We have performed many other experiments to realize the antiapoptosis mechanisms, such as caspase-3 detection. Unfortunately, no effect of DHI was observed on caspase-3. To clarify the virtual mechanisms of DHI, further investigations should be conducted, especially* in vivo*.

Unfortunately, not all indicators present a dose-dependent manner which might be because of the first-pass elimination of the liver, the role of P-glycoprotein in drug absorption, or the different compatibility effect of varied dosages. We proposed that low and middle concentrations of DHI protect the liver by alleviating the changes in enzymatic reaction and DHI with high concentrations attributed to anti-inflammation and antiapoptosis. Also, apoptosis may be the principal cause of death during AHF.

## 5. Conclusion

DHI attenuates D-GalN/LPS intoxication by exerting antioxidative, antiapoptotic properties and suppressing excessive production of TNF-*α* as well as the related inflammatory signaling cascade. This study provides evidence for widening the clinical application of DHI, especially its protective effect on AHF.

## Figures and Tables

**Table 1:** Effect of DHI on ALT, AST, and GST activities and MDA and TBil concentrations.

Group	Dose	ALT (U/I)	AST (U/I)	MDA (nmol/mg prot)	GST (U/mL)	TBil (mg/dL)
Control		62.14 ± 5.94	172.20 ± 29.91	0.06 ± 0.01	328.25 ± 35.46	0.22 ± 0.02
D-GalN/LPS		2541.86 ± 788.81^††^	452.76 ± 120.13^†^	0.30 ± 0.06^†^	527.85 ± 95.64^†^	0.55 ± 0.02^†^
NAC (mg/kg)	300	423.52 ± 62.77**	343.31 ± 51.20	0.29 ± 0.03	499.53 ± 93.41	0.46 ± 0.04*
DHI (mL/kg)	1.5	997.16 ± 227.95*	346.80 ± 80.59	0.20 ± 0.02	557.36 ± 79.22	0.38 ± 0.04*
	3	94.94 ± 15.51**	173.51 ± 25.51**	0.19 ± 0.01	302.69 ± 42.85*	0.28 ± 0.04**
	6	159.53 ± 51.86**	230.88 ± 40.15	0.20 ± 0.02	562.44 ± 70.96	0.51 ± 0.070

Data show mean ± SEM (*n* = 8).

^†, ††^ indicate significance from the control group at *P* < 0.05 and *P* < 0.01 probability level, respectively; ^∗, ∗∗^ indicate significance from the GalN/LPS group at *P* < 0.05 and *P* < 0.01 probability level.
